# DeepPath: overcoming data scarcity for protein transition pathway prediction using physics-based deep learning

**DOI:** 10.1039/d5sc08253f

**Published:** 2026-05-05

**Authors:** Yui Tik Pang, Lixinhao Yang, Katie M. Kuo, James C. Gumbart

**Affiliations:** a School of Physics, Georgia Institute of Technology Atlanta GA 30332 USA gumbart@physics.gatech.edu; b School of Chemistry and Biochemistry, Georgia Institute of Technology Atlanta GA 30332 USA

## Abstract

The structural dynamics of proteins play a crucial role in their function, yet many current deep learning methods chiefly yield high-resolution static snapshots of single conformations, with dynamics captured indirectly or at limited resolution unless paired with simulations or experiments. We present DeepPath, a physics-guided deep learning framework that rapidly predicts realistic protein transition pathways at atomistic resolution. Unlike conventional supervised learning approaches, DeepPath employs generative active learning (GAL) to iteratively refine its predictions, leveraging molecular mechanical force fields as oracles to guide pathway generation. We validated DeepPath on four biologically relevant test cases: AdK opening/closing, SHP2 activation, CdiB H1 expulsion, and BAM-complex gating, with DeepPath reproducing key transient interactions observed in previous studies. Notably, DeepPath also predicted an intermediate between the BAM inward- and outward-open states that closely aligns with an experimentally observed hybrid-barrel structure (TM-score = 0.91). This work highlights the potential of GAL for protein structure prediction.

## Abstract 2

DeepPath combines generative learning with classical force fields to discover physically realistic protein transition pathways.

## Graphical abstract

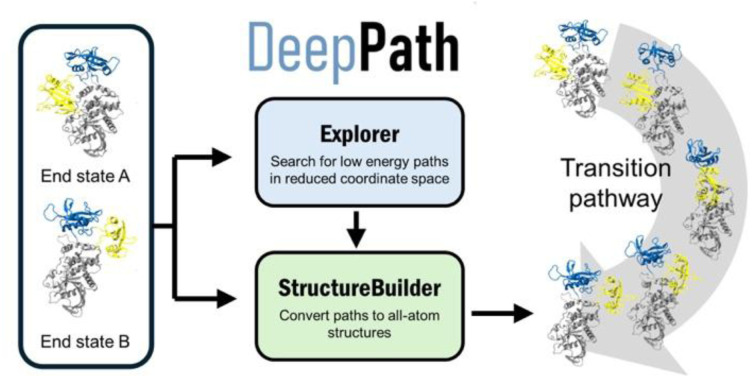

## Introduction

Proteins are fundamental to a plethora of biological processes, with their functions intrinsically linked to their three-dimensional structures. Accurate predictions of these structures have long been a central challenge in computational biology. The advent of deep learning techniques has revolutionized this field, particularly with the development of AlphaFold2 (AF2),^1^ swiftly followed by tools such as RoseTTAFold,^2^ ESMFold,^3^ AF3 (ref. 4) and others.^5–10^ These methods enable the accurate prediction of static protein structures from amino acid sequences, providing researchers with invaluable tools to investigate protein structures, functions, and interactions at an atomistic level.^11–15^ However, proteins are not static entities; they are dynamic molecules that undergo conformational changes essential for their biological functions. Capturing these transitions, not just endpoint structures, remains a key challenge.

Protein dynamics are essential to enable multiple functional states, ligand interactions, and participation in complex cellular pathways.^16–19^ Insights into these conformational changes are vital not only for basic research, but also for practical applications like drug discovery. Understanding conformational flexibility can guide the rational design of therapeutic molecules for efficacy and selectivity.^20,21^ MD simulations are traditionally the gold standard for studying protein dynamics *in silico*. They offer atomistic insights into molecular movements based on thermodynamic principles.^22^ However, these simulations are computationally demanding. Even with state-of-the-art supercomputers, MD typically achieves only nanoseconds to microseconds per day,^23–25^ far short of the millisecond-to-second timescales required for many biological processes. Enhanced sampling techniques^26–31^ and recent machine learning (ML) integrations^32–36^ aim to address this limitation, but they often remain computationally expensive and require highly specific input settings for different systems.

Building on the success of ML methods in static protein structure prediction, researchers have explored ways to leverage these tools to generate more diverse conformations.^37–41^ For example, masking coevolutionary signals in multiple sequence alignment (MSA) inputs^38–40^ or introducing alanine mutations^37,42^ were shown to induce some degree of conformational diversity in the generated structures.^42^ Other strategies include retraining models with additional structures derived from long-timescale MD simulations,^43^ adding diffusion-based or flow-matching-based auxiliary networks,^41,44^ or designing tailored architectures and training data for a smaller subset of proteins.^45–47^ BioEmu, which integrates several of these strategies, demonstrated the ability to generate ensembles of alternative backbone conformations for small proteins.^48^ These methods have achieved varying degrees of success, but none of them have obtained MD-level of diversity and structural resolution. A persistent challenge shared by all is the limited availability of training data for dynamical protein structures, which can hinder generalization, particularly for systems lacking prior dynamic data.^48^

In this paper, we experiment with a high-potential yet underexplored strategy called Generative Active Learning (GAL). Unlike traditional supervised learning, which passively learns from static, labeled datasets, GAL actively drives the learning process by iteratively generating candidates and refining its prediction based on feedback from oracles (*e.g.*, quantum chemistry simulations, docking scores, or physics-based models).^49,50^ This closed-loop optimization enables models to efficiently navigate vast design spaces, focusing computational and experimental resources on the most relevant regions. GAL has already been applied to areas like molecular chemistry^49,51,52^ and materials science^53^ for novel compound designs. These applications shared an important feature with the protein dynamics problem, where the available training data is still orders of magnitude short from filling in the vast configuration space, but well-developed physics-based models are available to predict the likelihood of reaching different states.

Here, we developed DeepPath, a novel deep learning architecture that leverages GAL to learn the transition pathways between two given protein structures by interacting with molecular mechanics (MM) force fields rather than from pre-computed pathway data. At the core of DeepPath is a two-module design: a generative-adversarial-network-(GAN)-based Explorer operating in reduced coordinates proposes transition pathways, while a dedicated Structure Builder reconstructs all-atom conformations, balancing computational speed and precision. To evaluate its capabilities, we tested DeepPath on a number of biologically relevant protein systems that exemplify key challenges in protein dynamics and large conformational changes. These systems range from soluble to membrane proteins, and from non-linear domain motion to substrate secretion. Specifically, we examined four protein transitions: domain opening/closing of adenylate kinase (AdK), activation of Src homology 2-containing protein tyrosine phosphatase-2 (SHP2), contact-dependent growth inhibition B (CdiB) helix 1 expulsion, and opening of the β-barrel assembly machinery (BAM) complex. We show that DeepPath efficiently explored the conformational spaces across all test cases, generating low-energy transition pathways within hours of training. Its predictions not only match existing data but also reveal novel conformations, broadening our understanding of these systems. Overall, these results underscore the potential of GAL as a scalable approach for predicting dynamic protein structures with all-atom resolution, even in the absence of large training datasets.

## Results

### Model overview

DeepPath introduces a GAL framework uniquely designed to overcome the scarcity of transition-state structural data for individual proteins. Instead of relying on static, precompiled datasets or pool-based active learning, DeepPath generates novel intermediates on the fly and refines them *via* interaction with a molecular mechanics (MM) energy minimizer as an “oracle”. This per-protein GAL loop, guided by force field energetics, allows DeepPath to autonomously expand its own training data, improving accuracy with each iteration without ever requiring a known pathway. To initiate the pathway exploration, short MD simulations are conducted of each of the input end states. These initial training data need not cover any of the transition states, but rather they give clues to the network about the relative mobility of each part of the protein. Then, DeepPath starts generating its own training data iteratively by constructing potential intermediate structures and validating their energies *via* an MM energy minimizer, slowly broadening the range of generated conformations while ensuring these structures remain realistic. Through this iterative process, DeepPath searches different possible transition pathways, expanding its training database until no alternative pathways with lower energies can be found.

**Fig. 1:**
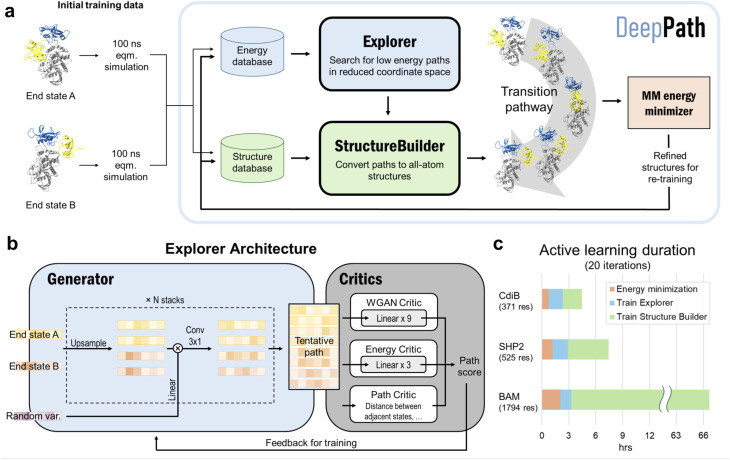
DeepPath overview. (a) GAL routine for training DeepPath. First, short MD equilibrium simulations are performed at each of the end state to generate some initial training data. This data is utilized to train the two modules of DeepPath, the Explorer and Structure Builder, which together produce all-atom structures along tentative transition pathways. Selected generated structures are refined with their corresponding energies determined using the MM energy minimizer. These refined structures are added to the training database to re-train DeepPath and gradually improve the reliability of the transition pathways generated as the training cycle continues. (b) The Explorer is a modified progressive GAN, with a combination of critics that work together to ensure the paths generated are realistic and energetically feasible. (c) Training duration for 20 iterations of GAL using one GPU for three protein test cases: CdiB, SHP2, and the BAM complex.

The architecture of DeepPath is modular by design, comprising two distinct but synergistic neural network components: the Explorer and the Structure Builder (Fig. 1a). The Explorer operates in a reduced coordinate space of carefully selected pairwise distances, enabling efficient exploration of the conformational landscape. It leverages a progressive GAN architecture,^54,55^ allowing coarse-to-fine pathway refinement while progressively increasing resolution over training iterations. The Structure Builder is a custom feed-forward network trained to reconstruct all-atom Cartesian coordinates from the Explorer's outputs. The choice of the reduced coordinate set is versatile, provided it can effectively differentiate between distinct protein conformations. To ensure the model's generality and physical interpretability, we have selected all residue–residue pairwise distances that show significant variation beyond a predetermined threshold as our reduced coordinates for this implementation.

The modular design of DeepPath is crucial to its success. The use of a reduced coordinate set isolates the Explorer from the high-dimensional coordinate space, which is characterized by a complex, uneven energy landscape that complicates the exploration and optimization of conformations. This separation of exploration from reconstruction avoids the pitfalls of directly learning in high-dimensional atomic spaces, a common source of instability in deep generative models for novel protein conformation generation. Additionally, this modular approach simplifies the overall network architecture, reduces the volume of training data required, and enables rapid production of valid transition pathways.

From our testing, DeepPath generates reliable transition pathways within 20 GAL iterations, which takes only a few hours on a single GPU for an average-sized protein (Fig. 1c) and achieves approximately 80% of the energy reduction observed at full convergence. For example, DeepPath computed the expulsion process of the H1 helix from the CdiB β-barrel, which has 371 residues, in less than 4.5 hours. DeepPath also proved scalable by accurately predicting the transition pathway between the inward-and outward-open states of the 1794-residue BAM complex, albeit requiring a longer computational time of 66.7 hours. A breakdown of the training time reveals that the majority of training cost increment is allocated to the training of the Structure Builder, the largest network within DeepPath. The use of the frame aligned point error (FAPE)^1^ in its loss function contributes to a computational complexity that scales quickly with the size of the protein, increasing from merely 2.1 hours for CdiB to 63.4 hours for the BAM complex. The time spent on energy minimization also increases with the complexity and size of the protein. In contrast, the training time for the Explorer remains largely constant across different protein sizes, highlighting the efficiency achieved through the use of reduced coordinate sets.

### Explorer

The core innovation of DeepPath resides in the Explorer, a GAN responsible of exploring the vast protein conformational space and constructing plausible transition pathways between the two specified input end states. These pathways are represented as a series of pairwise distance arrays (*x*) that encode each intermediate state along the pathway. We chose a progressive GAN as the fundamental architecture for the Explorer as we found its incremental nature to be highly compatible with our GAL protocol (Fig. 1b). The progressive GAN grows the network by starting the training at a low resolution, then adds new layers that model increasingly fine details as training progresses. We adopted this design for the Explorer by first training it to generate transition pathways represented by only four intermediate states, and then adding new layers to the generator and doubling the path resolution every five GAL training iterations until a sufficient resolution is reached. This staged learning approach allows the network to first capture a broad overview of the energy landscape before fine-tuning the pathways to minimize energy more effectively, balancing global exploration with local optimization.

Usually, GANs are composed of a generator and a critic, with the generator trained to generate outputs that are deemed realistic by the critic, and the critic trained to distinguish the generated outputs from the real ones. These two networks are trained against each other to improve each other's performance. Importantly, DeepPath trains the Explorer's generator not against ground-truth pathways but rather against a trio of specialized critics: a Wasserstein critic enforcing realism (*C*_WGAN_), an energy critic that approximates the MM-energy landscape (*C*_energy_), and a geometric path continuity critic (*C*_path_). This multi-critic training strategy^56^ enables stable generation of physically plausible transitions without requiring example pathways – a major departure from traditional supervised models. Together, the loss function for the generator is:1
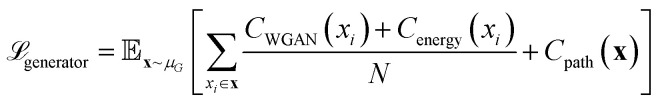
where *N* is the number of intermediate structures sampled from each generated path. *C*_WGAN_, implemented based on Wasserstein GAN (WGAN) with gradient penalty,^57^ is trained to distinguish between the pairwise-distance arrays produced by the generator against those from the structure database, guiding the generator to produce pairwise-distance arrays that resemble ones of the validated intermediate states. *C*_energy_ is trained to predict a structure's energy from its pairwise-distance representation, serving to guide the generator towards low-energy intermediate states. While *C*_WGAN_ is trained together with the generator, *C*_energy_ is trained independently at each GAL loop against the energy database. The three critics together ensure the generated pathways are realistic, low-energy, and continuous.

Outside of providing guidance for the generator, the *C*_WGAN_ and *C*_energy_ also serve another important purpose in the GAL process. Due to the high computational cost of performing energy minimization (compared to the rest of the training loop), only a fraction of generated intermediate structures were energy minimized and had their energies evaluated. Drawing parallels to the active learning criterion “query-by-committee”,^58^ we pick structures that obtain conflicting verdicts from *C*_WGAN_ and *C*_energy_. Specifically, if *C*_WGAN_ predicts the structure to be unrealistic but *C*_energy_ predicts it to be low energy, the structure is sent to have its energy evaluated. Conceptually, this criteria encourages structures that are rated novel and low-energy to be evaluated and added to the database, iteratively improving the training data quality and eventually leading to the generation of the most plausible transition pathways.

Together, these architectural choices allow DeepPath to generalize across protein sizes, topologies, and environments, from soluble domains to membrane-embedded β-barrels. The GAL loop ensures that DeepPath adapts to each protein individually, while the modular neural-network framework preserves interpretability and physical plausibility at every stage of generation.

### Test case 0: adenylate kinase

We first tested the network on adenylate kinase (AdK), a well-studied benchmark system whose open-closed conformational transition has been extensively characterized in molecular simulations. The AdK transition is commonly described using the distances between the LID-CORE and AMPbd-CORE domain centers, and both the associated free-energy landscapes^59^ and representative transition pathways^60^ in this space have been reported in previous work. Following these studies, we projected the DeepPath-generated intermediates onto the two-dimensional space defined by the LID-CORE and AMPbd-CORE distances. In this representation, the predicted pathway follows the same curved transition manifold observed in conventional simulation trajectories^60^ (Fig. S1), rather than a simple linear interpolation between the open and closed states. Importantly, the ordering of domain motions along the DeepPath pathway is also consistent with one of the transition trends previously reported for apo AdK: the AMPbd (NMP-binding) domain begins to open prior to the LID domain. Because the DeepPath test case also corresponds to the apo form of AdK, this sequential motion is consistent with previous simulation studies suggesting that the smaller and more flexible AMPbd domain tends to initiate opening motions prior to, or in concert with, the larger LID domain.^61,62^

### Test case 1: SHP2

To evaluate DeepPath in a real-world scenario, we applied it to a protein target characterized by a significant domain shift that is crucial to its pathogenic behavior. Src homology-2 domain-containing protein tyrosine phosphatase-2 (SHP2) is a protein target that has been implicated in cancer but was long considered undruggable.^63,64^ In a healthy cell, SHP2 typically remains in its closed state and only transitions into its open state upon binding with its partner protein. In cancerous cells, SHP2 can go from the closed to open conformation spontaneously, without binding of the partner protein.^65,66^ In addition, the protein sometimes has point mutations that bias it to the open state.^67,68^ The overactive SHP2 in the open conformation results in cancerous cell proliferation, tumor invasion, and metastasis.^69,70^ While there have been numerous small molecules developed against SHP2, none are FDA-approved, and all are designed to target the closed state.^66,71^ By understanding the transition from the closed to the open state, more effective therapeutics can be designed to inhibit this conformational transition and, thus, the pathogenesis of cancer.^69^ These conformational changes are particularly interesting for assessing whether DeepPath can generate pathways that are not strictly linear but instead rotational, and whether it can prevent the N-SH2 domain from colliding with the PTP domain. We were particular interested in the E76A variant due to the partially open state that had been previously reported.^67^

**Fig. 2:**
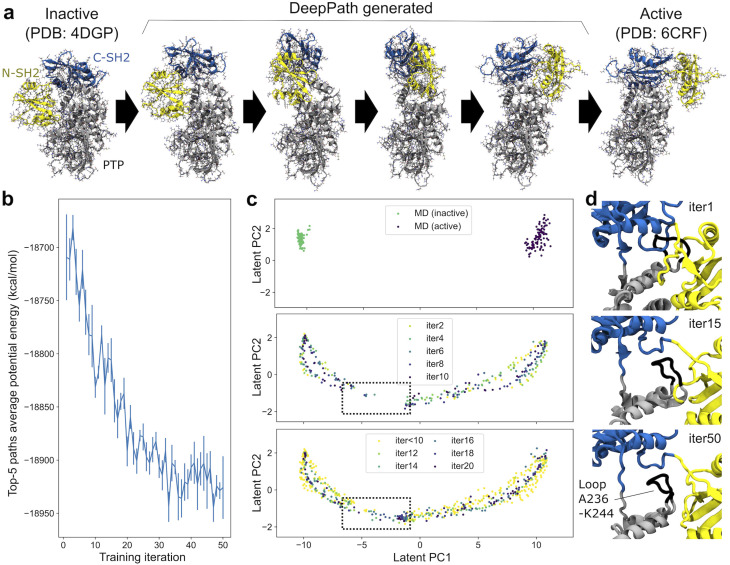
DeepPath predicted the transition paths between SHP2 active and inactive states. (a) The predicted transition pathway of SHP2 highlights the rotational movement of the N-SH2 domain (yellow) around the PTP domain (gray), accompanied by a rotation of the C-SH2 domain (blue). (b) The average potential energy of the top-five generated pathways decreases as training proceeds. (c) Training structures generated at different stages, projected onto the principal components of their reduced representations. Initial equilibrium simulations are shown in the top panel, GAL iterations 1–10 are shown in the middle panel, and iterations 11–20 in the bottom panel. The dotted square highlights the gap in the pathway that occurred early in training, which was successfully filled after 15 iterations of GAL training. (d) Close-up views highlights the challenge of generating structures in the region where the pathway initially breaks. Top: Before training, DeepPath predicts a structure with steric clashes around the A236–K244 loop (black) from the PTP domain. Middle: After 15 iterations of GAL, DeepPath lifts the C-SH2 domain, creating space for the A236–K244 loop to maneuver under the linker between the C-SH2 and N-SH2 domains, resolving the major steric clash between the A236–K244 loop and the C-SH2 domain. Bottom: At convergence, DeepPath completely resolves the steric clash issues, preventing the C-terminus from hooking into the A236–K244 loop.

DeepPath was initially trained on two 100 ns equilibrium MD trajectories, one originating from the inactive state and the other from the active state. Due to the short length of these trajectories, only a limited conformational space near each of the end states was sampled (Fig. 2c). Following the initial training, DeepPath was trained for 50 iterations using the GAL protocol. In each iteration, DeepPath evaluated the validity of selected structures it generated by determining whether their MM potential energy, computed by the energy minimizer, fell below an adaptive threshold. Once 32 valid structures were identified, they were incorporated into a database as part of the training dataset. Over the whole course of the GAL process, a total of 1600 new structures were added to the training set.

Fig. 2a shows the SHP2 transition pathway predicted by DeepPath. Consistent with previous studies,^65,66^ DeepPath identified a pivotal movement of the N-SH2 domain, which underwent a hinge-like swing around the PTP domain during the transition between the active and inactive states. Simultaneously, DeepPath predicted a rotation of the C-SH2 domain, positioned on top of the PTP domain. Interestingly, despite the large overall root-mean-square-deviation (RMSD) between the active and inactive states, the RMSD of each individual domain remains low throughout the predicted pathway (Fig. S2). This suggests that DeepPath preserved the local structural integrity of SHP2 during the transition, avoiding distortions that could compromise its functional state. Moreover, along the predicted transition pathway, DeepPath identified an intermediate conformation that closely resembles the partially open state reported by Tao *et al.*,^67^ notably recovering a critical contact between residues Y63 and E508 (Fig. S3).

Examining the progression of DeepPath during the GAL protocol, we observed that the average potential energy of the top-five generated pathways decreases gradually during the training and stabilizes at approximately −18 920 kcal mol^−1^ (Fig. 2b). This drop in energy can be explained by analyzing the structures generated by DeepPath during the training process. Early in the training, DeepPath showed an “understanding” that the N-SH2 domain must rotate around the PTP domain to transition between the active and inactive states. However, it initially lacked the ability to determine the optimal distance between the N-SH2 and PTP domains, resulting in steric clashes and higher energy barriers. Specifically, a loop (residues A236–K244) from the PTP domain clashed with both the N-SH2 and C-SH2 domains (Fig. 2d). When examining the validated structures in the training database, we noticed an early gap in the pathway, where all of the generated structures within the gap failed to obtain potential energies below the required threshold (Fig. 2c). However, DeepPath successfully generated valid structures that bridged this gap by the 14th iteration. It continued to produce more low-energy structures in subsequent iterations, eventually filling the gap entirely. The final predicted pathway reveals that DeepPath adjusted the C-SH2 domain, lifting it to create more space for the A236–K244 loop to pass underneath the linker between the C-SH2 and N-SH2 domains (Fig. 2d).

To assess the robustness of the DeepPath predictions, we examined the sensitivity of the results to the simulation setup used as the oracle. First, we repeated the SHP2 analysis using the AMBER ff14SB/TIP3P force field in addition to the CHARMM-based setup described above (Fig. S4).^72^ The projections in the latent coordinate space show that the generated structures progressively populate the same transition manifold as training proceeds, with the final pathways qualitatively similar between the two force fields. Although the AMBER-based runs required more iterations for the manifold to become continuous, this difference may reflect known variations in secondary-structure stability between force fields. In particular, the ff14SB/TIP3P combination tends to over-stabilize secondary structures,^73^ which could reduce hinge flexibility during early exploration.

We further investigated the role of the length of the initial MD trajectories used to generate the training data by repeating the analysis with trajectories of 20, 40, 60, and 80 ns (Fig. S5). When projected onto the same latent space defined by the 100 ns dataset (Fig. 2c), the 20 ns and 40 ns runs do not recover a well-defined transition manifold. In contrast, the 60 ns and 80 ns trajectories produce clear and continuous manifolds that closely resemble that obtained from the full 100 ns training trajectory. These results suggest that the quality of the learned transition manifold is influenced by the diversity of configurations sampled in the initial MD trajectories.

### Test case 2: CdiB

DeepPath's application extends beyond proteins with multiple stable conformations. In this section, we demonstrate its capability to study the expulsion of a domain from a transmembrane channel. The contact-dependent growth inhibition (CDI) system is a mechanism utilized by Gram-negative bacteria to suppress the growth of neighboring bacterial cells to reduce competition.^74^ As a member of the Two-Partner Secretion (TPS) family, the CDI system consists of two components: the CdiA toxin and the CdiB transporter. Here, we focused on CdiB, a β-barrel protein that inserts into the bacterial outer membrane, allowing CdiA to pass through its lumen and be displayed on the cell surface.^75–78^ However, in its resting state, this β-barrel is occluded by an N-terminal α-helix (H1); upon activation, H1 exits into the periplasm to enable CdiA secretion.^75,79–81^

**Fig. 3:**
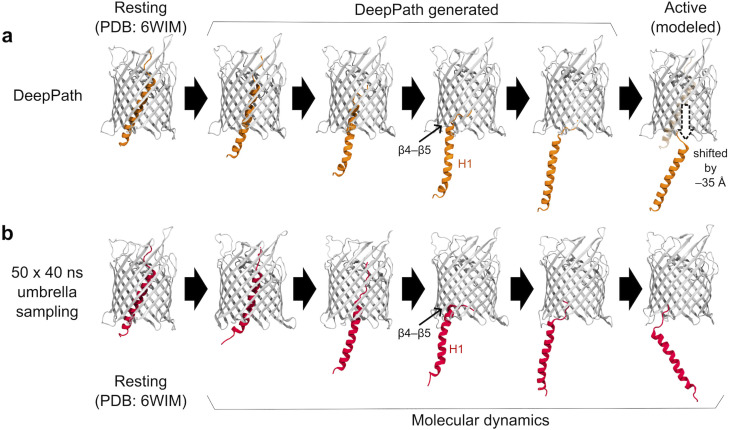
Exit pathway of the N-terminal α-helix (H1) in CdiB as predicted by (a) DeepPath and (b) REUS simulations from a previous study.^81^ DeepPath efficiently generated exit pathways that closely match the REUS-derived pathway, despite requiring significantly less computational time. Notably, DeepPath accurately captured the key transient interaction between H1 and β4–β5 during expulsion.

We used DeepPath to generate the exit pathway of the H1 helix and compared our results with a previous study^81^ that explored this process using steered MD (SMD) and replica exchange umbrella sampling (REUS). In the resting state, the upper half of H1 was tightly packed within the barrel lumen's narrower, more constricted region, limiting its movement. As expulsion progresses, H1 gradually slides downward, freeing itself from these constraints, and makes transient interactions with residues in the lower section of the β-barrel (Fig. 3b). Further examination of the trajectories revealed a particularly prominent interaction between H1 and the β4–β5 region.

Here, we assessed whether we can use DeepPath to recover this pathway with only the resting structure known. To construct the active state, we shifted H1 downward by 35 Å using VMD,^82^ positioning it entirely within the periplasmic space. Two 10 ns equilibrium MD simulations were run, one starting from the resting state and one from the modeled active state. Additionally, to aid DeepPath in generating an initial guess, a series of modeled intermediate structures were generated by incrementally displacing H1 downward at an interval of 0.35 Å. These additional structures underwent energy minimization but were not subjected to further simulations.

**Fig. 4:**
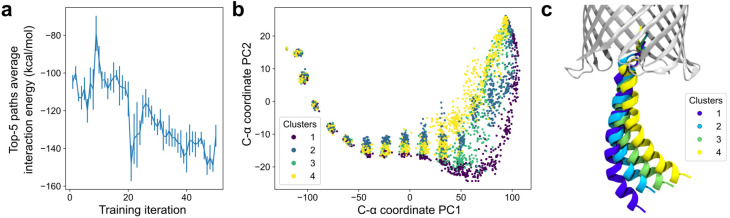
Diversity and energy refinement of DeepPath-generated H1 exit pathways. (a) Evolution of the interaction energy between H1 and the β-barrel over GAL training iterations shows an overall reduction in energy as DeepPath refined its predicted pathways. (b) PCA of the generated pathways based on the C-α coordinates of H1, with the β-barrel aligned. Each point represents an intermediate conformation, colored according to pathway clusters identified *via* path similarity analysis (PSA). (c) Representative conformations from each pathway cluster, illustrating structural variability in the periplasmic region while maintaining key contacts with the β-barrel.

The H1 exit pathway predicted by DeepPath after 50 iterations of GAL training highly resembles the one identified by the 2-µs aggregated REUS simulations (Fig. 3). The model predicted that H1 initially straightened itself slightly as it exits the narrow upper section of the β-barrel before moving downward, where it established transient interactions with the periplasmic end of β4–β5. Finally, it detached H1 from the β-barrel, reaching the modeled active conformation. Over the course of training, the interaction energy between H1 and the β-barrel gradually decreased (Fig. 4a). This trend suggests that DeepPath systematically explored alternative orientations of H1 and identified those leading to more favorable interactions. Additionally, when comparing the DeepPath output to the short equilibrium MD simulations, the DeepPath intermediates revealed distinct interactions between H1 and the β-barrel that are not present in the training data (Fig. S6).

Beyond reproducing the H1 exit pathway, DeepPath demonstrated the ability to generate diverse pathway predictions, particularly in regions where structural diversity does not come with an energy penalty. As observed in the REUS simulations, once H1 exited the β-barrel, its unconstrained portion could adopt a wide range of conformations. DeepPath also captured this change of flexibility. Looking at an ensemble of 256 transition pathways generated after training, all pathways were highly consistent when H1 was still confined within the barrel. However, as more residues emerged into the periplasmic space, the diversity among the generated pathways greatly increased while still maintaining key interactions, such as those between H1 and β4–β5 (Fig. 4b and c).

To further assess the extent of this diversity, we applied path similarity analysis (PSA)^83^ to cluster the pathway ensemble generated by DeepPath. We identified four distinct pathway clusters using a cluster distance cutoff of 28 in Ward criteria, indicating multiple viable exit pathways predicted by DeepPath. These clustering results align with the principal component analysis (PCA) of the H1 C-α coordinates, where the four pathway groups well-separated along the first two principal components (Fig. 4b). Notably, despite the structural variation, the average potential energies among all pathway clusters remained between −12 077 ± 7 to −12 072 ± 6 kcal mol^−1^, confirming that the pathway diversity was achieved without sacrificing structural integrity (Table S1). These findings suggest that DeepPath's prediction extends beyond a single transition pathway and instead approximates a transition tube, encompassing a diverse set of thermodynamically accessible routes at finite temperature.

### Test case 3: the BAM complex

To assess the scalability of DeepPath, we applied it to the BAM complex from *Escherichia coli*, a large multi-protein system with nearly 2000 total residues. The BAM complex consists of the transmembrane BamA β-barrel and the accessory proteins BamB–E. It plays a crucial role in the biogenesis of outer-membrane proteins (OMPs) in Gram-negative bacteria by facilitating their folding and insertion into the outer membrane.^84–86^ BAM adopts two distinct conformations: the inward-open and outward-open states.^87–91^ In the inward-open state, the lumen of BamA's β-barrel is accessible from the periplasm, allowing nascent OMPs to enter. In contrast, the outward-open state closes this passage while separating the first (β1) and last (β16) strands of the β-barrel, forming the lateral gate (LG), an entry point for nascent OMPs to integrate into the membrane. Recent structural studies revealed that during OMP insertion, BAM transiently forms a hybrid-barrel intermediate, where the substrate OMP uses BamA's β1 strand as a scaffold for folding.^92–96^ Here, we used DeepPath to predict the transition pathways between the inward- and outward-open states absent a substrate, training it solely on structural data from two 500 ns equilibrium MD simulations initiated from these two endpoint structures. Strikingly, DeepPath's predicted transition pathway revealed an intermediate conformation in agreement with an experimentally resolved hybrid-barrel structure of BAM interacting with EspP, a substrate OMP.^92^

**Fig. 5:**
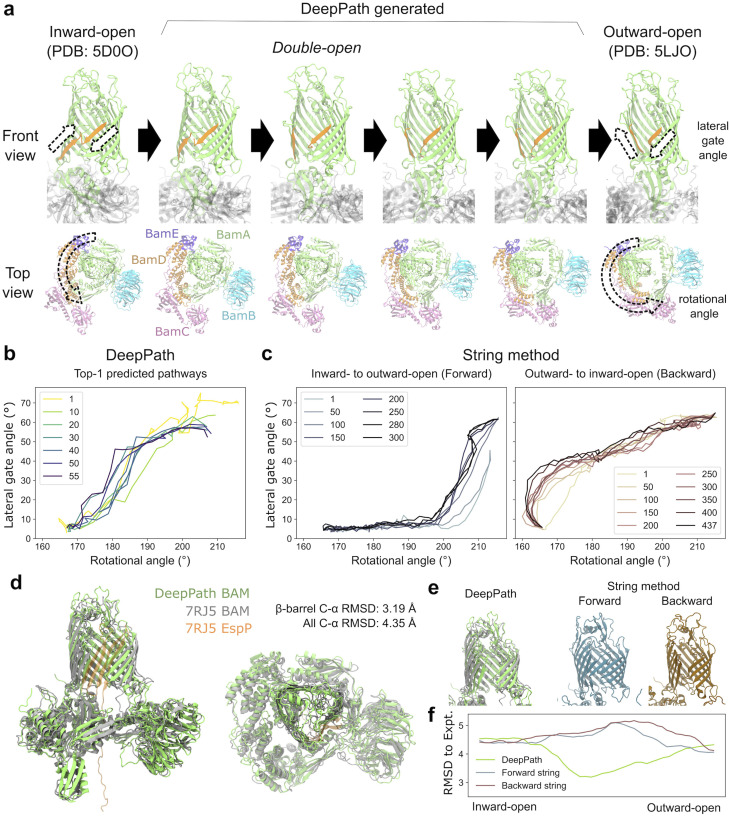
DeepPath-generated transition pathway of the BAM complex from the inward- to outward-open state and comparison to the string method. (a) Snapshots from the final iteration of DeepPath results showcasing the angular opening of the lateral gate (LG, orange), the movement of the P5 domain from left to right, and the counter-clockwise rotation of the periplasmic domains BamB–E as the LG opened. (b and c) Evolution of the transition pathways as represented by the LG angle and periplasmic domain rotational angle. (b) Top-1 pathways predicted by DeepPath. (c) Pathways obtained using SMwST, depicted for both inward-to-outward-open (forward) and outward-to-inward-open (backward) transitions. (d) The DeepPath-predicted “double-open” structure (green) overlapped with the cryo-EM structure of BAM/EspP hybrid-barrel intermediate (gray for BAM and orange for EspP; PDB: 7RJ5). Only shared residues of BAM were shown. (e) Comparison of the β-barrel structure predicted by DeepPath and SMwST during transition. (f) C-α RMSD between the predicted β-barrels and the hybrid-barrel experimental structure along the predicted transition pathways by DeepPath and SMwST.

From the DeepPath-predicted transition pathway, it is clear that conformational changes in BamA's β-barrel are tightly coupled with a counterclockwise rotation of the periplasmic domains (Fig. 5a). Between the inward- and outward-open states, DeepPath predicted a two-stage transition in the β-barrel: (1) LG opening and (2) base narrowing. During the first half of the transition, the β1 strand moved in sync with the rotation of the periplasmic domains. This motion quickly broke the backbone hydrogen bonds between β1 and β16 in the inward-open state, initiating LG opening. As β1 continued to straighten and peel away from β16, the LG reached its maximum opening width after the periplasmic domains rotated by approximately 15° (Fig. 5b). In the second half, the periplasmic domain rotation correlated with the narrowing of the β-barrel's base. The transition was complete once the periplasmic domains rotated by approximately 40° in total, at which point the base of the β-barrel constricted, fully sealing the periplasmic opening and the outward-open state was stabilized.

The DeepPath prediction suggests that BAM adopts an intermediate “double-open” state, where the BamA β-barrel lumen is accessible from both the periplasmic space and the membrane simultaneously. This observation is reminiscent of the hybrid-barrel intermediate, where both the LG and the periplasmic entrance must open to allow the nascent substrate OMP to pass through the BamA lumen and reach the LG as folding proceeds. To test whether the DeepPath-generated double-open structure corresponds to such an intermediate, we compared it to an experimentally resolved BAM/EspP hybrid-barrel intermediate structure (PDB: 7RJ5).^92^ Despite the absence of the substrate OMP in DeepPath's model, the alignment of the BamA β-barrel is remarkably high (Fig. 5d). The C-α RMSD between the predicted and experimental structures measured only 3.19 Å with a TM-score of 0.86. This structural agreement also extends to the membrane-inserting hydrophobic loops. Even without explicitly modeled membrane lipids, DeepPath correctly oriented the membrane-inserting hydrophobic loops, proving its capability to infer and preserve structural constraints purely from MD training data. Across all shared residues of the BAM complex, the C-α RMSD was 4.35 Å with a TM-score of 0.91. This demonstrates that DeepPath accurately captured the global structure, including the relative domain arrangement of the accessory proteins BamB–E (Fig. 5d).

As a comparison, we also applied the string method with swarms of trajectories (SMwST)^97^ to compute the same inward-to-outward-open transition pathway of the BAM complex. To generate the initial pathway estimates, we first ran two separate targeted MD (TMD) simulations: one pulling BAM from the inward- to outward-open state (forward) and another pulling it from the outward- to inward-open state (backward). These TMD-derived pathways were then used to seed two independent SMwST calculations. Each string was discretized into 51 equally spaced images, and 440–640 ps simulations were run per image, per iteration to evaluate the local energy gradients and update the pathway. The forward and backward strings were optimized for 300 and 437 iterations, respectively, accumulating to a total simulation time of 14.5 µs.

Overall, SMwST made significant progress in smoothing out abrupt transitions and reducing fluctuations, but major structural defects inherited from the original TMD pathways remained. Specifically, the forward TMD struggled to break the existing interactions that lock the LG in the inward-open state, while the backward TMD delayed widening the base of the β-barrel until the final stages of the pathway. Even after hundreds of SMwST iterations, remnants of these defects persisted in the optimized pathways. Notably, the forward and backward strings follow markedly different routes. In the forward string, the LG remains closed until the periplasmic domain has rotated for more than 30°, whereas in the backward string, LG closure occurred before any periplasmic domain rotation (Fig. 5c). Additionally, the forward transition retained a pronounced kink in the β1–β2 region, a distortion carried over from the initial TMD trajectory (Fig. 5e). Most importantly, neither the forward nor backward SMwST-generated pathways predicted the “double-open” state that DeepPath did. In fact, all intermediate structures generated by SMwST exhibited higher β-barrel C-α RMSD against the experimental hybrid-barrel structure (PDB: 7RJ5) than the starting end states, whereas DeepPath's predictions consistently achieved lower RMSD than the starting structures (Fig. 5f). This highlights DeepPath's superior predictive power in uncovering unseen intermediate structures accurately.

Comparing the optimization process of DeepPath with that of SMwST, we found that DeepPath converged much faster, both in terms of iterations required and computational cost. A key advantage of DeepPath is that, unlike traditional simulation-based methods, it is not constrained by directionality. Regardless of the simulation methods used to seed SMwST, the process must begin from one state and apply biasing forces to drive the system toward the other state. This approach unavoidably introduces distortions and human bias into the generated pathway. Once introduced, it would require hundreds of nanoseconds or even microseconds of equilibrium simulations to relax the structures and eliminate the defects, making it challenging for SMwST to recover physically realistic intermediates.

In contrast, DeepPath generates all intermediate conformations independently and simultaneously, without relying on biasing forces. Instead, it explores conformational space using a neural-network-driven approach that leverages the protein's intrinsic degrees of freedom and iterative energy evaluations, producing physically plausible initial guesses that require minimal structural corrections. Beyond better initial structure generation, DeepPath also demonstrated a strong ability to repair secondary structure distortions over optimization iterations. In early iterations, we observed twisting and breaking of native hydrogen bonds between BamA's β1 and β2, as well as disruptions in the secondary structure of extracellular loop 1. However, as the active learning iterations progressed, DeepPath gradually corrected these defects, ultimately producing secondary structures that align with the stable input conformations (Fig. S7). This refinement is accompanied by a systematic increase in β-strand content and a reduction of non-β assignments, with most residues either retaining or adopting β-strand character.

## Discussion

In this work, we introduce DeepPath, a GAL framework that constructs atomistic protein transition pathways by synthesizing and evaluating new training data on the fly, guided by physics-based energy evaluations. Unlike previous methods that require extensive protein structure datasets, DeepPath self-generates both its training set and predictions, representing a novel paradigm in protein structure modeling. DeepPath's core innovation lies in its combination of generative modeling, active learning, and physics-based oracles in a closed-loop framework. At each iteration, new structures are proposed by a GAN, scored by a set of critic networks, and refined using MM minimization. Through repeated structural refinement and exploration based on this energy evaluation, DeepPath gradually builds up the training dataset and efficiently produces atomic-resolution transition pathways within hours.

DeepPath demonstrates how GAL can be leveraged to exploit the physical and chemical knowledge embedded in MM force fields, training deep learning models to generate novel protein conformations even when curated examples of intermediates are scarce, which is a common bottleneck for models trained primarily on static structures or MD-derived sets. To achieve GAL, the Explorer has two additional critics alongside the standard discriminator network of GANs, namely the Energy and the Path Critics. Since there is a trio of critics, a balance of their loss weights is important for efficient learning and conformational space exploration. We found the ratio *C*_WGAN_ : *C*_energy_ : *C*_path_ = 1 : 1 : 10 quite effective for transitions with higher energy barriers (SHP2 and BAM), and 1 : 1 : 2 for cases that requires more pathway diversity (AdK and CdiB). The introduction of the Energy Critic network also enabled quick energy landscape exploration by minimizing expensive force-field energy computations and avoiding instability caused by direct energy-based optimization, a known limitation in earlier force-field-coupled generative models.^98^ However, the current implementation only evaluates protein potential energies and accounts for environmental factors using implicit solvent rather than explicitly modeling water molecules or membrane lipids. Future work could address this limitation by extending the Structure Builder module to construct other biomolecules including water, lipids, and nucleic acids, enabling more accurate computation of interaction energies with the environment.

One key architectural feature of DeepPath is its modular separation between conformational exploration and atomistic structure reconstruction. The GAN-based Explorer operates in a reduced coordinate space defined by pairwise C-α distances, enabling efficient global sampling of conformational space without directly navigating the high-dimensional and rugged all-atom landscape. The Structure Builder then reconstructs all-atom models from these reduced representations using physically motivated loss functions that enforce geometric plausibility, including FAPE, backbone bond length regularization, and steric clash penalties. This design allows DeepPath to integrate essential structural constraints into both training and inference while maintaining scalability across systems of varying size and topology; this degree of flexibility is not typically afforded by coordinate-space generative models. Together, these architectural decisions underlie DeepPath's ability to outperform traditional sampling methods in both efficiency and predictive power. While traditional approaches such as the string method required over 14.5 µs of simulation for the BAM complex, DeepPath produced realistic transition pathways in just 66 hours on a single GPU, matching an experimentally determined intermediate structure. This efficiency enables routine application to large, complex, or poorly characterized systems.

To assess generality, we evaluated DeepPath across four mechanistically distinct processes: conformational transition (AdK), domain rotation (SHP2), membrane-embedded helix expulsion (CdiB), and large multimeric-complex remodeling (BAM). In all cases, DeepPath identified physically plausible pathways, recovered known intermediates, and in the case of BAM, predicted a previously observed “double-open” hybrid-barrel conformation^92^ with a TM-score of 0.91. This was especially significant given the absence of the substrate OMP in DeepPath's input and output. Notably, all four systems were modeled using the same architecture and training strategy with minor adjustments, underscoring DeepPath's ability to generalize without system-specific retraining or architectural tuning. Beyond accuracy, DeepPath also demonstrated the ability to generate diverse transition pathways. In the CdiB test case, pathway clustering *via* PSA and PCA revealed four distinct transition route families and highlighted DeepPath's capacity to explore multiple low-energy transition pathways. While DeepPath successfully captures electrostatically driven transitions, its current implementation underrepresents entropic contributions and hydrophobic interactions. For instance, in the CdiB test case, hydrophobic contacts evident in the extended (10 µs) REUS simulations were not fully recovered. While current limitations stem from the simplified energy model, the modular nature of DeepPath provides a flexible foundation for incorporating more sophisticated scoring functions or oracle models in future iterations.

Although DeepPath is currently formulated to generate pathways between two predefined end states, in practice the method can still be applied when only one experimentally determined structure is available. In such cases, a plausible or approximate second state can be constructed to guide the exploration. DeepPath can then identify physically reasonable intermediates along the transition manifold before approaching the imposed endpoint. This behavior was observed in the CdiB system, where meaningful intermediate conformations were recovered even when the final configuration was only approximately specified. As expected, the reliability of the later stages of the pathway depends on how closely the hypothesized endpoint reflects the true target conformation.

It is important to note that DeepPath is designed to complement, not replace, MD simulations and enhanced sampling techniques, which remain the gold standard for capturing thermodynamic and kinetic properties. By rapidly generating plausible protein transition pathways, DeepPath can help guide MD sampling toward relevant regions and accelerate convergence in free energy calculations. From a physical perspective, the pathways generated by DeepPath can be interpreted as low-energy transition routes on the underlying potential energy landscape, obtained by combining geometric learning with iterative energy evaluation from the molecular-mechanics oracle. Related ideas have also emerged in approaches that reconstruct conformational pathways from cryo-EM classification or elastic network models, where the resulting motions correspond to dominant collective transitions in the system.^99,100^ Beyond its role in accelerating simulations, DeepPath also shows promise for drug discovery applications. Understanding conformational dynamics is critical for designing drugs that target allosteric sites or stabilize specific functional states of proteins. For example, many kinase inhibitors and molecular chaperone modulators rely on exploiting transient conformational states.^101–104^ DeepPath helps to identify these states, aiding in the development of more selective and effective therapeutics.

Beyond transition pathways, the GAL framework presented here could be extended to a variety of biomolecular structure prediction challenges, including ligand-induced conformational changes, protein folding pathways, and RNA structure prediction. These problems face an even greater scarcity of high-quality structural data, making GAL particularly valuable for efficiently navigating large conformational spaces and generating low-energy structures. The GAL framework introduced in this work effectively integrates ML with MM force fields, enabling structural prediction beyond the scope of the available training dataset. Future extensions of this approach could incorporate diffusion models or flow-based generative architectures, improving the ability to generate diverse structural ensembles, and better capturing the full conformational landscape of biomolecules beyond the current state-of-the-art supervised models.

## Methods

### Reduced coordinate sets

To lower GPU memory costs and increase exploration efficiency, the Explorer operated in a reduced coordinate space, while the Structure Builder converted the Explorer's outputs into all-atom structures in Cartesian coordinates of all heavy atoms. The reduced coordinates were defined as C-α distances between residue pairs that were in contact in one end state but not in the other. This set of residue pairs was identified using the following procedure: the pairwise C-α distances between all residues were calculated using MDAnalysis^105,106^ on the MD trajectories from both end states. Residue pairs were selected if their C-α distances fell below *ϵ*_A_ in at least one frame and exceeded *ϵ*_B_ in at least one other frame. For SHP2 and BAM, *ϵ*_A_ and *ϵ*_B_ were set to 6 Å and 15 Å, respectively. For CdiB, *ϵ*_A_ and *ϵ*_B_ were set to 7 Å and 15 Å, respectively.

### Explorer architecture

The Explorer is a generative adversarial network (GAN) with a generator and multiple critics. The generator's input is a pair of reduced coordinate sets, representing the end-state structures set for the pathway. Its output is a series of reduced coordinate sets, each representing an intermediate structure, and collectively they connect to form a predicted transition pathway. To facilitate this output structure, we adopted the architecture of a progressive GAN,^54,55^ with the output size doubling after each neural network block within the generator, *i.e.*, the first block produces a pathway with four intermediate states based on the two end states provided; and the second block builds on top of it and creates a pathway with eight intermediate states; and so on. The training procedure was also progressive: during the first five active learning iterations, only the first block was trained; in the next five iterations, the first two blocks were trained; and so forth. In this study, the maximum number of blocks used was four, with the final block added at the 16th active learning iteration. This resulted in an output size of 32 intermediate structures, after which no further blocks were added. The generator was trained to reduce the total loss calculated by all the critics (eqn (1)).

The Explorer consists of three critics: WGAN, Energy, and Path Critics. To ensure every point on the pathway is being optimized, the WGAN and Energy Critics evaluate the loss based on 32 intermediate structures sampled at random intervals along an interpolated pathways formed by linear interpolating between the generated structures. Path Critic evaluates the pathway quality loss using the generated structures directly without interpolation.

The WGAN Critic (*C*_WGAN_) is a multi-layer perceptron (MLP) with eight hidden layers, each containing 128 neurons with LeakyReLU activations. Its input is a single intermediate structure in reduced coordinates, and it outputs a “realism” score for the input structure. The loss function for this critic follows the WGAN with gradient penalty,^57^ with an extra regularization term 
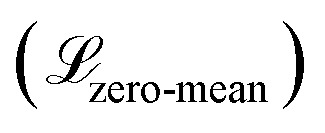
 to enforce a zero-centered score distribution:2
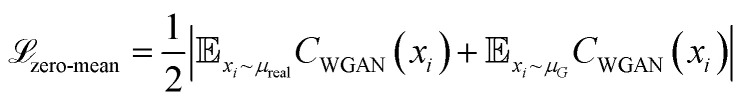


The WGAN Critic is trained together with the generator.

The Energy Critic (*C*_energy_) is an MLP with two hidden layers, with 128 and 64 neurons, respectively, and ReLU activations. Its input is also a single intermediate structure in reduced coordinates, and it outputs the probability for the input structure to fall into each of the ten energy bins. We found predicting energy labels to be more tolerant to data noise, compared to predicting the energy value directly. Categorical cross-entropy was used as the loss function. It is trained independent of the generator. To make the Energy critic robust against out-of-distribution samples produced by the generator, we employed ensemble knowledge distillation. Three independent teacher models were first trained on ground-truth labels from randomly drawn subsets of the energy-labeled structures. Then, soft pseudo-labels were produced on all generated structure using this ensemble of teacher models. Finally, a student model was trained on a mix of ground-truth labels and soft labels:3
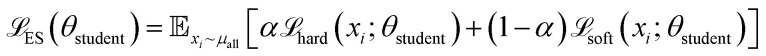
4
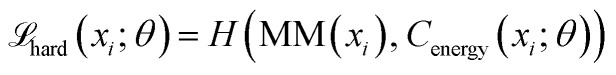
5
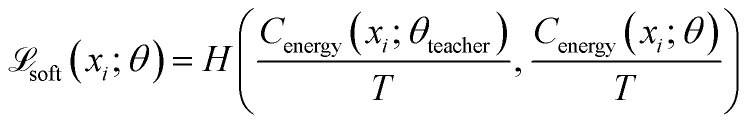
where *H*(*y*_1_, *y*_2_) is the cross entropy, and MM(*x*_*i*_) is the one-hot energy label of structure *x*_*i*_ measured by the MM energy minimizer, *α* = 0.1 and 0 for samples with and without ground-truth labels, respectively, and the temperature *T* = 3.

The Path Critic is algorithmic-based and does not contain any trainable parameters. It computes the mean squared error (MSE) between the vector difference of adjacent structures in reduced coordinate space generated by the generator and the baseline one formed by linear interpolation between the two end states:6
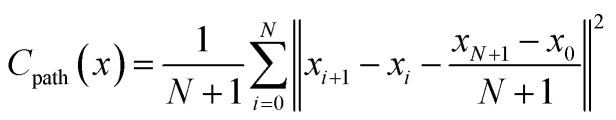
where *N* is the number of intermediate structures sampled from each generated path. In addition, there are two more auxiliary terms in Path Critic to enhance the quality and diversity of the generated structures: (1) a triangular-well potential to penalize predicted reduced coordinates, which are set to C-α distances, from going below 3.5 Å, and (2) a linear penalty that applies to any two structures within the same batch when their median absolute deviation (MAD) in reduced coordinate falls below 0.5 Å. Together, these components guide the model to generate pathways that remain structurally realistic, energetically feasible, and geometrically continuous. In our framework, the WGAN term restricts structures to the conformational manifold supported by the MD training data, the energy critic biases sampling toward low-energy regions of the landscape, and the continuity terms enforce gradual structural changes along the pathway, thereby maintaining the statistical and physical relevance of the predicted transitions.

### Structure Builder architecture

The Structure Builder is a custom feed-forward network that converts structures from reduced coordinates to all-atom Cartesian coordinates. It consists of a repeating block (Algorithm 1) that progressively optimizes the so-called backbone frames and the dihedral angles of the sidechains, a reduced protein representation that has been proven effective in AF2.^1^ The block is repeated five times. In the end, the Cartesian coordinates of all heavy atoms are computed from the backbone frames and sidechain rotation components generated by the last block.
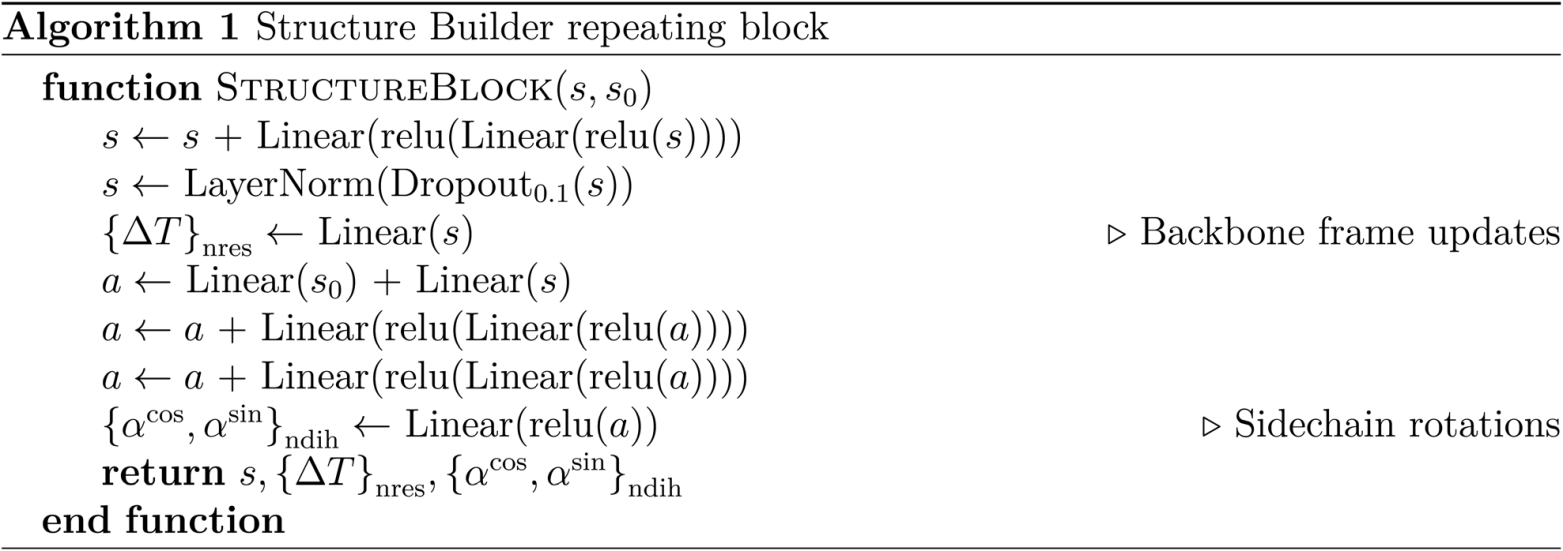


The training loss for Structure Builder is based on both the intermediate outputs of each repeating block and the final structure it predicts. During the initial training, the loss consists of frame-aligned point error (FAPE) of all predicted intermediate backbone frames 
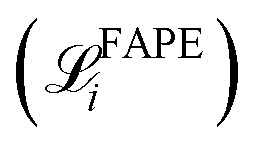
 and the final all-atom structures 
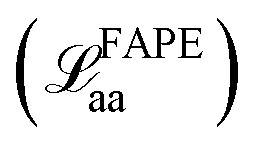
, and MSE of the cosine and sine of all predicted sidechain angles 
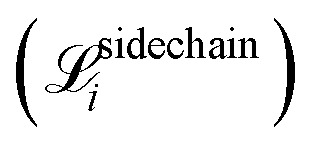
. In subsequent training inside the active learning loop, three extra terms are added. First, a flat bottom L1-loss on the amide bond length 
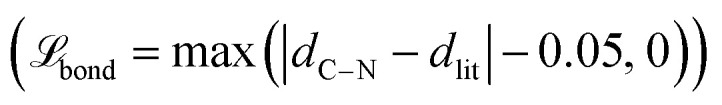
 to penalize bonds that deviate from the theoretical length (1.334 Å) by more than 0.05 Å. Second, a flat bottom potential that penalizes atoms that getting closer than 1.2 Å from each other 
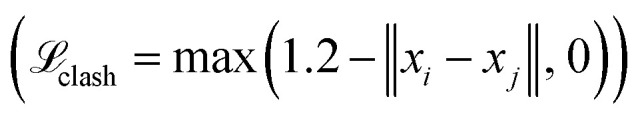
. Optionally, a sigmoid of the final all-atom structure RMSD 
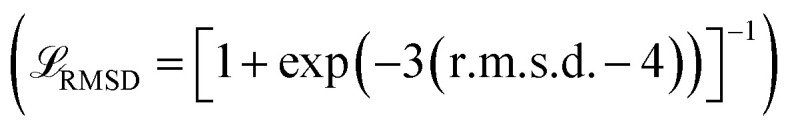
 can be added to enforce the global domain arrangement. Among the four test cases, it is only turned on when generating the BAM complex transition paths. The total losses during the two stages are:7
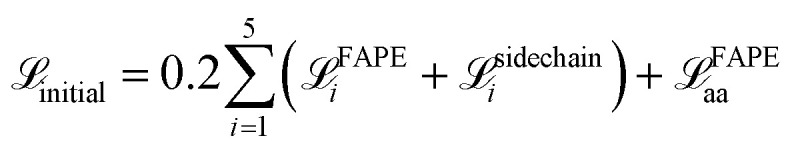
8
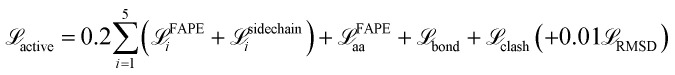


The learning rates during the initial and subsequent trainings are 0.001 and 0.0001, respectively.

### Generative active learning training procedure

DeepPath was trained using a generative active learning (GAL) framework to refine its prediction of transition pathways, minimizing the reliance on pre-existing datasets. The training procedure is described as follows:

1. Initial training: a set of initial structural training data was prepared (see DeepPath initial training set generation). These structures were then energy-minimized and translated into reduced representations. The Structure Builder was trained on the minimized structures until it can reproduce the protein domain arrangements at both end states with reasonable accuracy. It took 1000, 500, and 2000 training epochs for SHP2, CdiB and the BAM complex, respectively. The Energy Critic was trained on the potential energies of the minimized structures for 1000 epochs without knowledge distillation.

2. Explorer GAN training: the generator and WGAN Critic of the Explorer were trained jointly in an adversarial manner (see Explorer architecture). The generator was trained for 50 to 2000 epochs, with the number of epochs reducing as the training database grows in size. For each epoch the generator was trained, the WGAN critic was trained for two epochs.

3. Candidate structures generation and selection: a batch of 256 tentative transition pathways were generated by the Explorer. Intermediate structures were sampled and selected from these pathways with two selection processes. First, intermediate structures with high predicted energies from the pathway with the lowest total predicted energy were selected. This is to help DeepPath close gaps that might exist in its generated pathways. Second, intermediate structures predicted to be low energy by Energy Critic but deemed non-realistic by WGAN Critic were selected. This strategy draws parallels to the “query-by-committee” active learning selection criteria^58^ to encourage sampling of structures DeepPath is uncertain of. A total of 32 structures were selected in each training loop for SHP2 and CdiB, while 24 structures were selected for the BAM complex.

4. Oracle consultation: the selected intermediate structures were converted into all-atom structures by Structure Builder. These structures were then energy minimized through three steps. First, the backbone atoms were locked in place and only the sidechain atoms were minimized. Next, the structures were minimized again with the backbone locks removed but replaced with harmonic restraints on specific pairwise C-α distances. These included the distances forming the reduced coordinate space in the Explorer to ensure the newly generated structures adhere to the learned transition pathway. Hence, the equilibrium positions of these restraints were set to match the reduced coordinates originally output by the Explorer. In addition, harmonic restraints were used on C-α distances that were deemed constant (below 9 Å and has standard deviation below 0.4 Å throughout the original MD trajectories) to ensure existing residue–residue interactions were retrained. The force constant of these restraints was set to 20 kcal mol^−1^ Å^−2^. Finally, the structures were minimized once again without any restrictions. The potential energies of the final minimized structures were recorded. If their energies were lower than the median energy plus one MAD of the existing structures in the training database, the structures were accepted. However, if fewer than half of the final minimized structures fulfilled this limit, the half of the structures with the lowest energies were accepted instead. The accepted structures were added to the training database.

5. Retraining Structure Builder and Energy Critic: the Structure Builder and Energy Critic were retrained using the updated database. The Structure Builder was trained for 5 to 50 epochs, decreasing as the database grows. The Energy Critic was trained using ensemble knowledge distillation (see Explorer architecture). First, each of the three teacher models was trained for 2000 to 10 000 epochs. Then, the student model was trained for 1000 to 3000 epochs.

6. Iterations and convergence: steps 2–5 were repeated until no significantly lower-energy pathways and no novel conformations were identified. Such criteria were reached after 50 GAL iterations for SHP2 and CdiB and after 55 iterations for the BAM complex.

All the energy evaluation and minimization within the GAL iterations were performed using OpenMM 7.7.^107^ The CHARMM36m protein force field^108^ was used for SHP2 and the BAM complex, and CHARMM36 (ref. 109) was used for CdiB. In addition, for SHP2 and CdiB, Onufriev-Bashford-Case GBSA model^110^ with the GB^OBC^II parameters was used to implicitly model solvents, with the solute and solvent dielectrics set to 1.0 and 78.5, respectively. An implicit solvent model was not used for the BAM complex. The reported training time was based on our testing using a single NVIDIA GeForce RTX 4090 for the SHP2 and CdiB and an NVIDIA A100 80GB PCIe for the BAM complex. The main user-provided inputs and the key training parameters used in DeepPath are summarized in Table S2. All models are trained with Adam optimizer^111^ using the default settings from Tensorflow 2.14 (ref. 112) unless otherwise specified.

### Protein model constructions

For SHP2, the inactive and active states were built from publicly available structures. The inactive state was built from PDB ID: 4DGP while the active state was built from PDB ID: 6CRF. Missing loops and residues were added from the AlphaFold predicted structure. To build the mutant E76A, the mutate function in VMD was implemented through Psfgen. SHP2 was built in a TIP3P waterbox with dimensions 100 Å × 100 Å × 100 Å and 0.15 M NaCl.

The CdiB and the BAM complex models were prepared and adopted directly from previous studies.^81,92^

### Molecular dynamics (MD) simulations

MD simulations were performed using NAMD 2.12–2.14 (ref. 113) and NAMD 3.0b.^23^ The CHARMM36m protein force field^108^ was used for the SHP2 simulations. The CHARMM36 protein^109^ and lipid force fields^114^ were used for the CdiB and BAM complex simulations. All simulations used TIP3P water.^115^ A uniform 4 fs time step was employed through the use of hydrogen mass repartitioning (HMR).^116,117^ Long-range electrostatics were calculated at each time step using the particle-mesh Ewald method.^118^ A short-range cutoff for Lennard-Jones interactions was set at 12 Å, with a switching function beginning at 10 Å. Bonds involving hydrogen atoms were constrained to their equilibrium lengths, employing the SETTLE algorithm for water molecules and the SHAKE algorithm for all others. Unless otherwise specified, the temperature and pressure were fixed at 310 K and 1 atm, respectively, using Langevin dynamics and piston,^119^ respectively.

#### DeepPath initial training set generation

For SHP2 and CdiB, equilibrium simulations were run from the end-state structures for 100 ns and 10 ns, respectively. 100 frames extracted from each trajectory at regular intervals were used as initial training data for DeepPath. For the BAM complex, the beginning 500 ns of the equilibrium simulations from Wu *et al.*^92^ were used directly, providing 50 frames at 10 ns intervals for each end state. For CdiB, 100 additional structures were created using VMD^82^ by shifting H1 downward bit by bit at 0.35 Å intervals from the resting structure (PDB: 6WIM), until H1 was 35 Å below the original resting position.

#### String method with swarm of trajectories (SMwST)

SMwST calculations^97^ were seeded by targeted MD (TMD) trajectories, where external forces were applied to decrease RMSD between the current coordinates in the simulation and the target structure linearly. Two TMD simulations were run, each to seed an independent SMwST calculation: the forward TMD drove the inward-open state (PDB: 5D0O) to the outward-open state (PDB: 5D0Q), and backwards one drove the outward-open state (PDB: 5LJO) to inward-open state (PDB: 5D0O). An external force with an elastic constant of 200 kcal mol^−1^ Å^−2^ were applied to all shared C-α atoms to drive these transformation. Before the force and the RMSD were computed, the BamA β-barrel was aligned to that of the starting structure. A 2 fs time step was used without the application of HMR. Other simulation parameters were identical to the above description. The simulation length for each TMD simulation was 100 ns.

Following TMD, additional constraint simulations were run to ensure the systems fully reached the target structures and the two pathways' end states were identical. The C-α atoms of the BAM complex were constrained to the outward-open state (PDB: 5LJO) and inward-open state (PDB: 5D0O) starting from the last frame of the forward and backward TMDs, respectively. The constraining force constant was gradually increased from 0.2 kcal mol^−1^ Å^−2^ to 15 kcal mol^−1^ Å^−2^ over 17.4 ns of simulation time.

Frames were extracted from the TMD simulations to form the initial strings. Each string was composed of 51 equally-spaced images, putting each neighboring image roughly 1.5 Å apart from each other. The two end images of both forward and backward strings were originally fixed at the first- and last-frame structures from their respective TMD simulations. At iterations 56 and 117 of the forward and backward strings, respectively, the end-state structures were changed to those from the additional constraint simulations.

SMwST requires users to define the collective variables (CVs) that describe the conformation change along the transition pathway being studied. To minimize human intervention, we selected Cartesian coordinates of every other C-α atom in the BAM complex as the CVs. An adaptation of SMwST for high-dimensional CVs from Ma and Schulten^120^ was used in this study. The forward and backward strings were optimized for 300 and 437 iterations, respectively, accumulating to a total simulation time of 14.5 µs.

At each iteration, 20 replicas (swarms) of short trajectories (20 ps) were launched from each image. The average drift among these trajectories was computed, and the image is updated accordingly. A smoothing algorithm was employed to redistribute the images such that they remain equidistant from each other.^120^ A short constraint simulation (40–240 ps) was run to pull the closest swarm trajectories' last frame to the new designated location of each image.

## Author contributions

J. C. G. and Y. T. P. conceptualized the project. Y. T. P., L. Y., and K. M. K. curated and analyzed the data. All authors contributed to writing, reviewing, and editing the draft.

## Conflicts of interest

Y. T. P., K. M. K., and J. C. G. are co-inventors on a provisional patent application (18/441606 submitted by the Georgia Institute of Technology) covering the methodological advances described in this article. They are also stockholders of Atomistic Insights, a company that aims to develop the inventions described in this manuscript. The remaining author declares no competing interests.

## Supplementary Material

SC-017-D5SC08253F-s001

## Data Availability

Trained models are provided at https://huggingface.co/andrewytp/deeppath. Supplementary information (SI): detailed pseudocodes, additional analysis, and parameter tables. See DOI: https://doi.org/10.1039/d5sc08253f.
