# Supplementary material for: DeepPath: overcoming data scarcity for protein transition pathway prediction using physics-based deep learning
Source: Chem Sci. 2026 May 5;17(24):12055–73. doi: 10.1039/d5sc08253f (PMC13164416; doi:10.1039/d5sc08253f)
Supplement: SC-017-D5SC08253F-s001 [file SC-017-D5SC08253F-s001.pdf]

Supplementary Information (SI) for Chemical Science.
This journal is © The Royal Society of Chemistry 2026

Supporting Information

DeepPath detailed implementation

Notations

$\{...\}_{\text{size}}$ represents a list of arrays. If the size is omitted, it remains the same as last defined. All densely connected layers (Linear) have 256 neurons unless otherwise specified. $\{RC\}$ is a list of structures along the generated path in reduced-coordinate representation. $\{x^{\text{aa}}\}$ is a such list in Cartesian coordinates. They can also be represented in backbone frames ($\{T\}$) and sidechain dihedral angles components ($\{\alpha^{\cos}, \alpha^{\sin}\}$).

Explorer

Algorithm S1 Explorer

function EXPLORER($\{RC^{\text{endpoint}}\}$, n_{conv}, $n_{\text{latent}} = 8$)
  $\{s_0\} \leftarrow \{\emptyset\}_2$
  for $i = 0$ to 1 **do** ▷ Shared weights
    $s_0[i] \leftarrow \text{relu}(\text{Linear}_{128}(RC_i^{\text{endpoint}}))$
  end for
  $z \leftarrow \text{LayerNorm}(\text{RandomNormal}())$
  for $i = 1$ to n_{latent} **do**
    $z \leftarrow \text{LeakyReLU}_{0.2}\ (\text{Linear}_{128}(\text{z}))$
  end for

  $\{s\} \leftarrow \{s_0\}$
  for $i = 1$ to n_{conv} **do**
    $\{s\} \leftarrow \text{Repeat}(\{s\}, 2)$ ▷ Upsample
    $s \leftarrow s \cdot \text{LeakyReLU}_{0.2}(\text{Linear}_{128}(\text{z}))$ ▷ Modulate
    $s \leftarrow \text{Concat}(s_0[0], s, s_0[1])$ ▷ Pad with endpoint representations
    $s \leftarrow \text{LeakyReLU}_{0.2}\ (\text{Conv1D}_3\ (s))$ ▷ Convolution
    $s \leftarrow s/(\text{stddev}(s) + \epsilon)$ ▷ Normalization
  end for
  $\{RC\} \leftarrow \text{softplus}(\text{Conv1D}_1\ (\text{s}))$
  $\{RC\} \leftarrow \text{Concat}(RC_0^{\text{endpoint}}, \{RC\}, RC_1^{\text{endpoint}})$ ▷ Pad with endpoints
  return $\{RC\}$
end function

Structure Builder

Algorithm S2 Structure Builder

function STRUCTUREBUILDER(RC, $n_{\text{layer}} = 5$)
  $s_0 \leftarrow \text{relu}(\text{Linear}_{512}(RC))$
  $s \leftarrow \text{Linear}(s_0)$
  $\{T_0\} \leftarrow \{\mathbb{1}_4\}_{\text{nres}}$ ▷ Initialize T as identity matrices
  for $i = 1$ to n_{layer} **do**
    $s, \{\Delta T\}_{\text{nres}}, \{\alpha_i^{\cos}, \alpha_i^{\sin}\}_{\text{ndih}} \leftarrow$ STRUCTUREBLOCK(s, s_0)
    $T_i \leftarrow T_{i-1} \cdot \Delta T$ ▷ Apply incremental transformation
  end for
  $\{T^{\text{aa}}\}_{\text{natom}}, \{x^{\text{aa}}\}_{\text{natom}} \leftarrow$ ALLATOMCOORDINATES$(\{T_{\text{nlayer}}\}, \{\alpha_{\text{nlayer}}^{\cos}, \alpha_{\text{nlayer}}^{\sin}\})$
  return $\{T^{\text{aa}}\}, \{x^{\text{aa}}\}, \{T_i\}, \{\alpha_i^{\cos}, \alpha_i^{\sin}\} \quad \forall i \in [1, n_{\text{layer}}]$
end function

Refer to Algorithm 1 in the Main Text for the StructureBlock().

Algorithm S3 All-atom coordinate constructor

function AllAtomCoordinates($\{T\}_{\text{nres}}$, $\{\alpha^{\cos}, \alpha^{\sin}\}_{\text{ndih}}$, $\{\text{dih\_type}\}_{\text{ndih}}$)
    $(\alpha^{\cos}, \alpha^{\sin}) \leftarrow (\alpha^{\cos}, \alpha^{\sin}) / \|\alpha\|$ ▷ Normalize $\alpha^{\cos}$ and $\alpha^{\sin}$
    $T^{\alpha} \leftarrow \begin{pmatrix} 1 & 0 & 0 & 0 \\ 0 & \alpha^{\cos} & -\alpha^{\sin} & 0 \\ 0 & \alpha^{\sin} & \alpha^{\cos} & 0 \\ 0 & 0 & 0 & 1 \end{pmatrix}$ ▷ Construct T base on predicted rotation angles

    $\{T^{\text{dih}}\} \leftarrow \{\emptyset\}_{\text{ndih}}$
    for $i = 1$ to n_{dih} **do**
        if dih_type[i] is 'ψ' or 'χ_1' **then**
            $T^{\text{base}} \leftarrow T[\text{res}[i]]$ ▷ Use backbone as base
        else
            $T^{\text{base}} \leftarrow T^{\text{dih}}[i-1]$ ▷ Use the preceding sidechain fragment as base
        end if
        $T^{\text{dih}}[i] \leftarrow T^{\text{base}} \cdot T^{\text{lit}}[\text{dih\_type}[i]] \cdot T^{\alpha}[i]$
        ▷ Combine base and local transformations
    end for

    $\{x^{\text{aa}}\} \leftarrow \{\vec{0}\}_{\text{natom}}$
    for $i = 1$ to n_{natom} **do**
        $T^{\text{aa}} \leftarrow T^{\text{dih}}[\text{dih\_from\_atomID}[i]]$
        $x^{\text{aa}}[i] \leftarrow T^{\text{aa}} \cdot x^{\text{lit}}[i]$ ▷ Apply corresponding T^{dih} to atom coordinates
    end for

    return $\{T^{\text{aa}}\}, \{x^{\text{aa}}\}$
end function

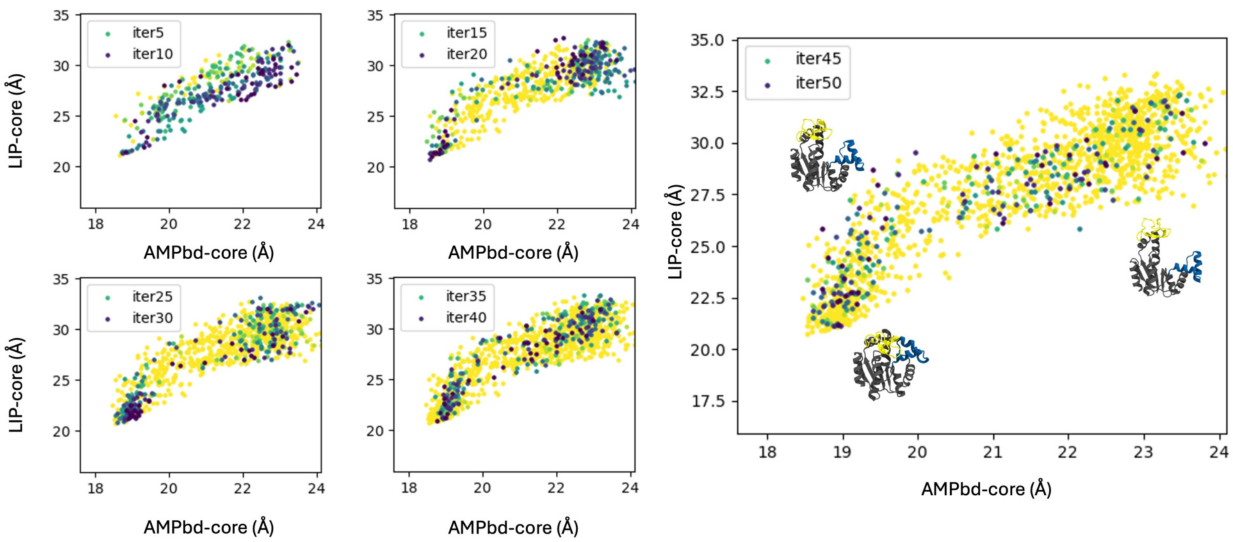

Figure S1: Iterative filling of the transition manifold for the ADK open-to-closed transition.

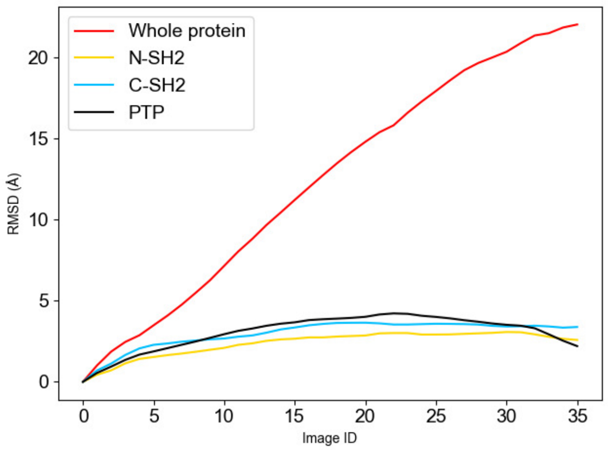

Figure S2: RMSD of the full SHP2 protein and its individual domains, computed relative to the predicted inactive state (image ID 0), for all structures along the predicted transition pathway. Only heavy atoms were used in the RMSD measurement.

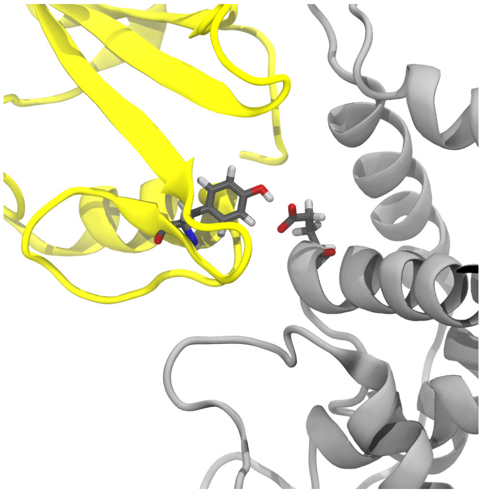

Figure S3: DeepPath identified the critical contact between residues Y63 and E508 of SHP2.

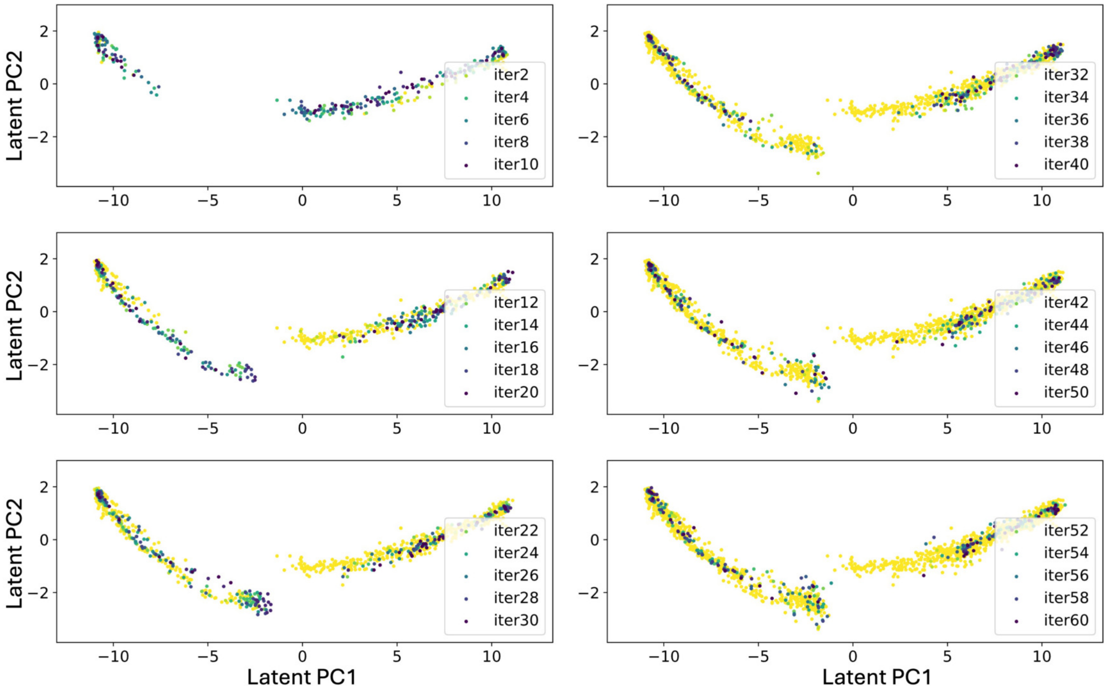

Figure S4: Latent space projections of DeepPath-generated structures during training using the AMBER force field. Each panel shows PCA projections (PC1 vs PC2) of the pair-distance representation, with colors indicating different training iterations.

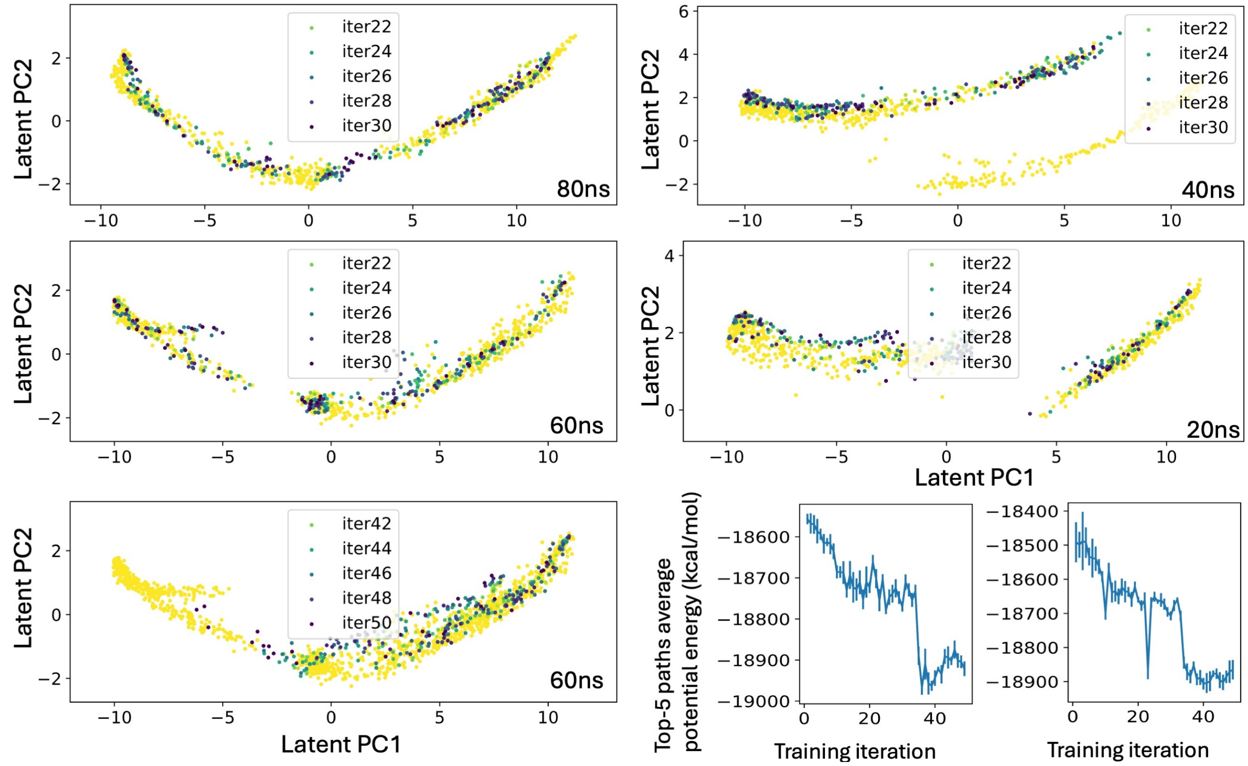

Figure S5: Effect of the length of the initial MD trajectory on DeepPath training for the SHP2 system. Generated structures are projected onto the same latent PCA space defined by the 100 ns dataset to enable direct comparison. Results are shown for training datasets derived from 80 ns (top left), 40 ns (top right), 60 ns (middle left), and 20 ns (middle right) MD trajectories, with points colored by training iteration. The shorter trajectories (20 ns and 40 ns) do not recover a continuous transition manifold, whereas the 60 ns and 80 ns datasets produce clearer transition pathways. The bottom-left panel shows later training iterations for the 60 ns dataset (iterations 40–50), illustrating the progressive filling of the transition manifold. The bottom-right panels show the evolution of the average potential energy of the top five predicted pathways during training for the 80 ns (left) and 60 ns (right) datasets, where the energy decreases as the model discovers lower-energy intermediates along the transition pathway.

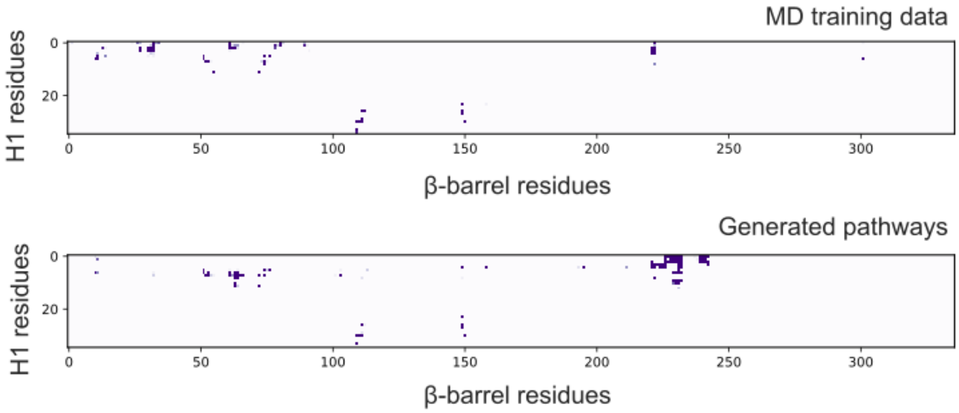

Figure S6: Contact maps between C-α atoms of the CdiB β-barrel and H1 helix using 7 Å as cutoff distance, identical the cutoff used for reduced coordinate construction. Residue pairs that are colored in the upper panel are include as part of the reduced coordinate set used by DeepPath. The lower panel shows the contacts exhibited by generated pathways, which includes contacts that are not part of the reduced coordinate set.

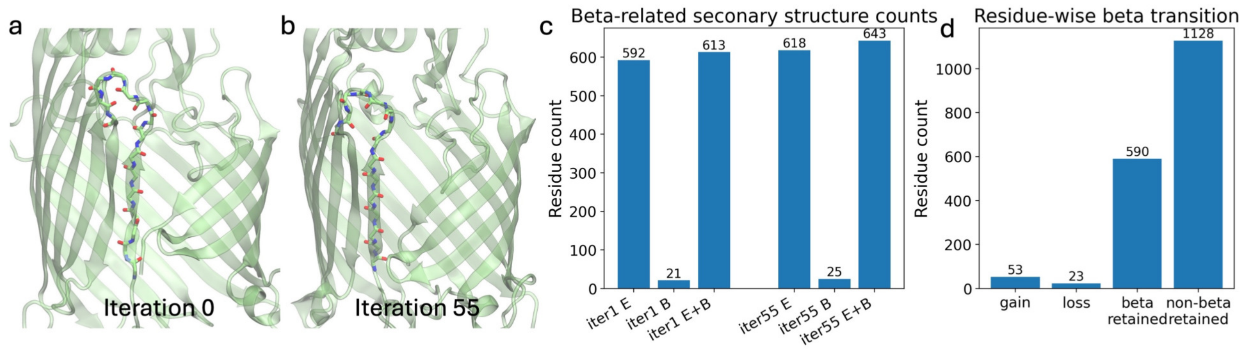

Figure S7: Correction of the BAM lateral gate secondary structure during DeepPath active learning. (a,b) Representative structures from early (iteration 1) and late (iteration 55) stages, showing the refinement of the lateral gate region toward a β-strand conformation. (c) Quantification of β-related secondary structure elements, showing an increase in β-content and a reduction of non-β assignments over training. (d) Residue-wise summary of secondary structure transitions, indicating that most residues either retain or adopt β-strand character, with relatively few losses.

Table S1: Energy statistics of CdiB path clusters found by DeepPath.

Cluster ID	No. of paths	Total energy (kcal/mol)	Interaction energy (kcal/mol)
0	52	-12072 ± 6	-128 ± 5
1	47	-12076 ± 6	-129 ± 7
2	59	-12076 ± 6	-136 ± 6
3	98	-12077 ± 7	-139 ± 5

Table S2: Inputs and key parameters used in DeepPath modeling. All notations follow the definitions in the Methods section.

Type	Item	Description	Value
**Inputs**			
Input	Protein force field	Protein force field used for energy evaluation in OpenMM	CHARMM36m (AdK, SHP2, BAM), CHARMM36 (CdiB)
Input	Solvent force field	Implicit solvent force field used for energy evaluation in OpenMM	$GB^{OBC}II$ (AdK, SHP2, CdiB), None (BAM)
Input	MD trajectories	Initial MD trajectories per end-state used to construct reduced coordinates and training data	1 ns (AdK), 10 ns (CdiB), 100 ns (SHP2), 500 ns (BAM)
**Reduced coordinate definition**			
Parameter	ϵ_A	Distance cutoff for defining residue contacts	6 Å (SHP2, BAM), 7 Å (CdiB)
Parameter	ϵ_B	Distance cutoff for defining non-contacts	15 Å (all systems)
**Explorer generator loss components (Eq. 1)**			
Loss	C_{WGAN}	WGAN critic score	weight = 1.0

Type	Item	Description	Value
Loss	C_{energy}	Energy critic score	weight = 1.0
Loss	C_{path}	Path continuity loss based on deviation from linear interpolation	weight = 10.0 (SHP2, BAM), weight = 2.0 (AdK, CdiB)
Loss	Clash penalty	Triangular-well potential penalizing distances below 3.5 Å	weight = 1.0
Loss	Diversity penalty	Linear penalty for structures with MAD below 0.5 Å in reduced coordinates	weight = 1.0
**Explorer critics loss components**			
Loss	$\mathcal{L}_{\text{WGAN}}$	WGAN critic loss	weight = 1.0
Loss	$\mathcal{L}_{\text{GP}}$	Gradient penalty	weight = 10.0
Loss	$\mathcal{L}_{\text{zero-mean}}$	zero-centered score regularization	weight = 1.0
Loss	$\mathcal{L}_{\text{ES}}$	Energy critic student network loss	$\alpha = 0.1$, $T = 3$
**Structure Builder loss functions**			
Loss	$\mathcal{L}_i^{\text{FAPE}}$	Frame-aligned point error for intermediate backbone frames	weight = 0.2
Loss	$\mathcal{L}_{\text{aa}}^{\text{FAPE}}$	FAPE of final all-atom structure	weight = 1.0
Loss	$\mathcal{L}_i^{\text{sidechain}}$	MSE of sidechain dihedral angles	weight = 0.2
Loss	$\mathcal{L}_{\text{bond}}$	Flat-bottom L1 loss enforcing amide bond length	weight = 0 (initial), weight = 1.0 (active learning)
Loss	$\mathcal{L}_{\text{clash}}$	Steric clash penalty for atoms closer than 1.2 Å	weight = 0 (initial), weight = 1.0 (active learning)

Type	Item	Description	Value
Loss	$\mathcal{L}_{\text{RMSD}}$	Sigmoid RMSD restraint on final structure	weight = 0.01 (BAM during active learning), weight = 0 (otherwise)
**Training parameters**			
Parameter	Learning rate	Adam optimizer learning rate	0.001 (initial), 0.0001 (active learning)
Parameter	GAL iterations	Number of active learning cycles	50 (AdK, SHP2, CdiB), 55 (BAM)
Parameter	Selected structures	Number of structures added per iteration	32 (AdK, SHP2, CdiB), 24 (BAM)
